# A Prospective, Multicenter Study of Laboratory Cross-Contamination of *Mycobacterium tuberculosis* Cultures

**DOI:** 10.3201/eid0811.020298

**Published:** 2002-11

**Authors:** Robert M. Jasmer, Marguerite Roemer, John Hamilton, John Bunter, Christopher R. Braden, Thomas M. Shinnick, Edward P. Desmond

**Affiliations:** * San Francisco General Hospital Medical Center and University of California, San Francisco, California, USA; † San Francisco General Hospital, San Francisco, California, USA; ‡ Santa Clara Valley Medical Center, San Jose, California, USA; § Solano County Public Health Laboratory, Vallejo, California, USA; ¶ Centers for Disease Control and Prevention, Atlanta, Georgia, USA; ** California Department of Health Services, Berkeley, California, USA

**Keywords:** *Mycobacterium tuberculosis*, laboratory cross-contamination, DNA fingerprinting, RFLP typing

## Abstract

A prospective study of false-positive cultures of *Mycobacterium tuberculosis* that resulted from laboratory cross-contamination was conducted at three laboratories in California. Laboratory cross-contamination accounted for 2% of the positive cultures. Cross-contamination should be a concern when an isolate matches the genotype of another sample processed during the same period.

 Culture remains the reference standard for diagnosis of disease caused by *Mycobacterium tuberculosis*. However, false-positive results can be caused by cross-contamination of cultures in the laboratory, e.g., when *M. tuberculosis* bacilli are transferred from one specimen to another specimen that does not contain viable bacilli (1–10). Historically, determining whether false-positive results are caused by laboratory cross-contamination has been difficult because of the lack of specific strain identification and nonsystematic criteria. False-positive cultures for *M. tuberculosis* have important implications for clinical management of patients. Many patients are treated on the basis of the results; therefore, patients can be exposed to unnecessary, potentially toxic, and costly treatment. Genotyping of *M. tuberculosis* strains has become the standard method for determining whether isolates are clonal (11–14). This technique, in combination with a review of clinical and radiographic data, allows a determination of the incidence of laboratory cross-contamination of *M. tuberculosis* cultures. In this study, we used predefined criteria to investigate possible laboratory cross-contamination *of M. tuberculosis* cultures and prospectively determine its incidence in an effort to find methods to decrease the occurrence of cross-contamination.

## Methods

### Study Laboratories and Patients

 This study was conducted by staff of the Microbial Diseases Laboratory, California Department of Health Services, Berkeley, California, which is one of seven participants in the National Tuberculosis Genotyping and Surveillance Network of the Centers for Disease Control and Prevention. Participating California laboratories included those at the San Francisco General Hospital, Santa Clara Valley Medical Center (San Jose), and Solano County Public Health Laboratory (Vallejo). The study was conducted from January 1, 1998, to June 30, 1999.

### Laboratory Methods

*M. tuberculosis* isolates from all sources underwent IS*6110*-based DNA restriction fragment length polymorphism (RFLP) analysis (11) if they were 1) the patient’s first *M. tuberculosis–*positive culture derived from a sample cultured in the participating laboratory; 2) cultured from a specimen collected >30 days after an *M. tuberculosis* culture–negative specimen was obtained; or 3) cultured from a specimen collected >90 days after the start of appropriate anti-tuberculosis (TB) therapy. When five or fewer bands were present, the isolates underwent secondary genotyping with a RFLP analysis based on a polymorphic GC-rich sequence (12–14).

RFLP pattern images were entered into a database and compared to identify isolates with matching genotypes. Any of the following cultures were considered potentially cross-contaminated and underwent further investigation: 1) the first *M. tuberculosis–*positive culture for a patient whose isolate had a genotype that matched that of another isolate cultured or used in the participating laboratory 2 days before or after the potentially cross-contaminated culture; 2) an *M. tuberculosis* culture from a specimen obtained >30 days after the collection of an *M. tuberculosis* culture-negative specimen that had an isolate with a genotype different from that of any previous isolate from the same patient; or 3) an *M. tuberculosis* culture, from a specimen collected >90 days after the start of appropriate anti-TB therapy, in which the isolate had a genotype different from that of any previous isolate from the same patient.

Patients with specimens meeting the above criteria and for whom potential source isolates were identified (i.e., their isolates had a genotype that matched that of a potential source isolate) underwent further investigation. The investigation included a review of all clinical data and radiologic studies, if applicable, and possible epidemiologic connections between patients with potential source and contaminated specimens. Clinical data included prior history of TB, results of tuberculin skin tests, treatment of latent TB infection, symptoms of present illness, results of diagnostic evaluations for TB, and alternative diagnoses. Personnel in laboratories where the potentially cross-contaminated specimens were processed also investigated potential sources of cross-contamination within their facilities.

### Determination of Cross-Contamination

 A final determination of laboratory cross-contamination was made after a panel of experts reviewed each case. The panel comprised three genotyping network investigators from sites other than California. Panel members met once at the conclusion of the study and reviewed all information from laboratory and medical records, epidemiologic investigations, and genotyping images (upon request). Cases were presented to the panel members in person by one of the authors (RJ). Panel members independently recorded their conclusions on whether cross-contamination was likely, as well as their final diagnosis, and submitted these results to the senior investigator of the study (ED).

## Results

 Review showed that similar methods for specimen processing were used in all three participating laboratories. However, one laboratory used a common flask for dispensing decontaminant reagent and phosphate buffer rather than an individual tube or pipette for each specimen.

 During the study period, 21,835 specimens were submitted for mycobacterial culturing at the three laboratories. Of these, 988 (4.5%) from 296 different patients were positive for *M. tuberculosis*. Twenty-seven had only a single positive culture. Of these, specimens from 10 patients met criteria for an investigation of possible laboratory cross-contamination (Table).

**Table:** Clinical and laboratory characteristics of patients suspected of having false-positive cultures for *Mycobacterium tuberculosis*^a^

Patient	Lab	Specimen type	Initial or follow-up specimen	Sputum smear result	Clinical signs and symptoms	Comments	Panel decision	Final diagnosis
1	1	Sputum	Initial	Negative	36-yr-old man with AIDS hospitalized with cough, dyspnea, right lower lobe infiltrate; he improved with trimethoprim and sulfamethoxazole alone	Genotype of isolate matched that of another patient whose specimen was processed same day	Cross-contamination	Bacterial pneumonia
2	2	Sputum	Initial	2+ Positive	28-yr-old man with HIV infection with fever, cough, right lower lobe infiltrate; improved with ceftazidime	Genotype of isolate matched that of another patient hospitalized on the same ward	Mislabeled specimen	Bacterial pneumonia
3	2	Sputum	Initial	Negative	31-yr-old woman hospitalized with cough, left lower lobe infiltrate, and leukocytosis; chest radiograph showed improvement with clindamycin and ofloxacin	Genotype of isolate matched that of another patient whose specimen was processed same day	Cross-contamination	Bacterial pneumonia
4	2	Sputum	Initial	Negative	44-yr-old man hospitalized with cough and right lower lobe infiltrate; improved with levofloxacin before anti-TB therapy initiated	Genotype of isolate matched that of another patient whose specimen in the BACTEC instrument immediately preceded the case patient	Cross-contamination	Lung abscess
5	2	Sputum	Follow-up	Negative	68-yr-old woman newly immigrated from China with cough; chest radiograph showed bi-apical fibronodular changes	Genotype of second isolate >30 days later did not match that of initial isolate or any other isolate in database	Mislabeled specimen or mixed infection	Bacterial pneumonia versus TB with mixed infection**]**
6	2	Sputum	Initial	4+ Positive	82-yr-old man with cough, fever; chest radiograph showed chronically increased right mid-lung interstitial markings	Genotype of isolate matched that of another patient hospitalized on the same ward	Mislabeled specimen or cross-contamination	Bacterial pneumonia
7	2	Sputum	Initial	Negative	27-yr-old woman with upper respiratory symptoms; chest radiograph was normal	Genotype of isolate matched that of another patient whose specimen was processed same day	Cross-contamination	Upper respiratory tract infection
8	2	Sputum	Initial	Negative	44-yr-old man with hemoptysis and known pulmonary metastases of squamous cell carcinoma of trachea; chest radiograph showed three large cavities with air-fluid levels; bronchoscopy culture grew *H. influenzae*, and patient improved on antibiotics alone	Genotype of isolate matched that of another patient whose specimen was processed same day	Cross-contamination	Lung abscess
9	2	Broncho-alveloar lavage	Initial	Negative	55-yr-old woman hospitalized with dyspnea and left lower lobe infiltrate; she improved with broad-spectrum antibiotics alone	Genotype of isolate matched that of another patient whose specimen was processed same day	Cross-contamination	Bacterial pneumonia
10	3	Sputum	Follow-up	Negative	34-yr-old homeless man with HIV infection, fever, and cervical lymphadenopathy; chest radiograph showed left lung nodule	Genotype of initial isolate matched that of another homeless TB patient; follow-up specimen (35 days later) had a unique genotype	TB	TB

^a^Lab, laboratory; TB, tuberculosis; *H. influenzae, Haemophilus influenzae*.

 After the panel’s review, laboratory cross-contamination was identified as the cause for positive culture results for six patients (2% of all patients with cultures positive for *M. tuberculosis*) (Table). Rates were similar at two of the three laboratories, ranging from 2.8% in both Laboratories 1 (1 of 36 patients) and 2 (5 of 179 patients) to 0% (0 of 81 patients) with a culture positive for *M. tuberculosis* in Laboratory 3. At Laboratory 2, a common flask was used for dispensing reagents during the study period. One of the cross-contamination incidents (involving Patient 4) probably resulted from a malfunctioning broth-culturing system (BACTEC 460) (Becton Dickinson Microbiology Systems, Sparks MD). Patient 4’s specimen was in the BACTEC instrument immediately after another patient’s specimen (not processed on the same day), and genotypes of the two isolates matched. Cultures from two patients (Patients 7 and 9) were cross-contaminated from the same source patient during sample processing. All six of the laboratory cross-contamination incidents occurred with the initial rather than follow-up specimens for mycobacterial culture. Five of these six patients were treated for TB. Of the remaining four patients whose isolates were suspected of being cross-contaminated in the laboratory, one had a false-positive culture attributed to specimen mislabeling by a health-care provider; one had either a mislabeled specimen or mixed infection; one had active TB; and one had either a mislabeled specimen or a cross-contaminated specimen (Table). Having only a single positive culture was highly associated with laboratory cross-contamination (p<0.001, Fisher exact test).

## Discussion

 Laboratory cross-contamination was the cause for a positive culture result in 2% of all patients with *M. tuberculosis–*positive cultures. Cross-contamination accounted for one fifth (22%) of patients having only one culture positive for *M. tuberculosis*. Of the six patients who had cross-contaminated cultures, five were treated unnecessarily with multiple anti-TB medications.

 The rate of cross-contaminated cultures in our study is similar to the rates in previous studies of *M. tuberculosis* cultures. Most population-based studies found rates of 0.9% to 3.5% (1–8). However, such studies were retrospective and did not assess the extent of the problem in different types of clinical mycobacteriology laboratories. In this study, we used predefined criteria, which were based largely on DNA genotyping, to identify suspected cases of laboratory cross-contamination prospectively in an effort to correct factors associated with its occurrence. Our study included all clinical specimens submitted during a 1.5-year period to one county public health laboratory and two county hospital laboratories. This study was possible because of the large databank of RFLP results conducted as part of being a member of the genotyping network.

Multiple factors can cause false-positive cultures, including contaminated clinical equipment (e.g., bronchoscope), clerical errors, and cross-contamination that occurs in the laboratory. The last category can be caused by batch processing, transfer of viable bacilli from the sample needle of a broth-culturing system, e.g., BACTEC (15), a faulty exhaust hood (4), and contamination from species identification procedures such as the niacin production test (6).

Five of the six cross-contamination incidents were in a single laboratory. In four of these five cases, contamination probably occurred when reagents were dispensed with a common flask. Previous studies have reported that the step of adding the phosphate buffer was likely to have been the source of the cross-contamination (1,6,9). This procedure was later discontinued on the basis of the results of this study. None of the laboratories used positive control cultures.

 As in other studies, we found that a single positive culture for *M. tuberculosis* was a sensitive but nonspecific marker for detection of a false-positive culture, since most patients (78%) with a single positive culture had TB. In a New York study (7), 12 (44.4%) of 27 patients with a single positive culture had a false-positive culture. These findings suggest that clinicians and laboratorians should be increasingly suspicious of a single false-positive culture. Additional specimens should be collected in cases of a single false-positive culture and the patient evaluated carefully for TB and other illnesses; the laboratory should also retain the isolate and others processed that day for genotyping. Because all specimens that met the inclusion criteria in our study were from the respiratory tract, we cannot draw any conclusions about the rate of cross-contamination of nonrespiratory specimens (e.g., cerebrospinal or pleural fluid). Nor can we draw any conclusions about a single positive culture when only a single specimen is submitted to the laboratory, as is often the case with nonrespiratory specimens.

 Clinical judgment is also important in raising suspicion about cross-contamination. TB classically is accompanied by symptoms of prolonged cough, fever, weight loss, and night sweats, but other diseases such as bacterial pneumonia can cause these symptoms. Therefore, no specific clinical criterion alone can be used to definitively state that TB is present. However, an inconsistent clinical course or absence of symptoms should certainly raise suspicion that cross-contamination may have occurred (3,8,16). A determination regarding the presence of cross-contamination requires a thorough evaluation of a patient’s symptoms and clinical course as well as laboratory evaluation, with additional specimens obtained if only a single culture is positive as described above. Genotyping should be performed if cross-contamination is suspected on the basis of an inconsistent clinical course or the presence of only one positive culture (8).

 Our assessment of the rate of cross-contamination did not include private laboratories; thus, our results may not be generalizable to all types of clinical laboratories. In addition, our methods depended on identifying, obtaining, and genotyping an isolate from a positive source culture; thus, we may have underestimated the true rate of laboratory cross-contamination.
